# Percutaneous cryoablation of abdominal wall endometriosis: a systematic literature review of safety and efficacy

**DOI:** 10.1186/s13244-024-01823-4

**Published:** 2024-11-22

**Authors:** Sylvain Bodard, Leo Razakamanantsoa, Ruben Geevarghese, Julianne O’Gorman, Anthony Dohan, Clement Marcelin, François H. Cornelis

**Affiliations:** 1 Department of Radiology, Memorial Sloan Kettering Cancer Center, New York, NY 10065 USA; 2 Weill Cornell Medical College, 1300 York Avenue, New York, NY 10065 USA; 3 University of Paris Cité, Department of Radiology, Necker Hospital, 149 rue de Sèvre, 75015 Paris, France; 4 Sorbonne University, CNRS UMR 7371, INSERM U 1146, Laboratoire d’Imagerie Biomédicale, 75006 Paris, France; 5 Sorbonne University, Department of Radiology, Tenon Hospital, 75020 Paris, France; 6 Saint-Antoine Research Center (CRSA), INSERM, CNRS, Sorbonne University, F-75012 Paris, France; 7 Department of Radiology, Hopital Cochin, 75014 Paris, France; 8 Department of Radiology, CHU Bordeaux, Place Amélie Raba Léon, 33076 Bordeaux, France

**Keywords:** Endometriosis, Cryoablation, Pain, Interventional radiology, Imaging

## Abstract

**Purpose:**

To investigate over 10 years the safety and efficacy of percutaneous cryoablation for the treatment of abdominal wall endometriosis (AWE).

**Methods:**

A systematic review was conducted of literature published between March 2014 and March 2024. Inclusion criteria focused on treatment efficacy studies, while exclusion criteria targeted case reports and studies lacking pertinent outcome data. Methodological quality was assessed using the Newcastle-Ottawa Scale for cohort studies.

**Results:**

A total of eight studies were included. Local pain scores decreased from a median of 8/10 (interquartile range (IQR) 7–9) on the visual analog scale to 1/10 (IQR 0–2) at the last follow-up (*p* < 0.0001). Median complete local pain response rates ranged from 80% to 100%, with median local pain-free survival rates reaching 76.8% (IQR 55.3–83.8) at the longest follow-up. Notably, no patient reported a post-procedure pain score higher than that they reported pre-cryoablation. The studies indicated minor complications in 3.5 to 11% of cases, with major complications occurring in less than 2% of cases, graded following the guidelines of the Society of Interventional Radiology.

**Conclusion:**

In the last decade, percutaneous image-guided cryoablation has offered consistent results and appears to be a promising, minimally invasive option for AWE treatment. Prospective trials are now essential to establish cryoablation as a new standard in patient care for AWE.

**Critical relevance statement:**

Over a decade-long study, percutaneous cryoablation has proven to be a safe and effective minimally invasive treatment for abdominal wall endometriosis, significantly reducing pain with minimal complications.

**Key Points:**

- Percutaneous cryoablation significantly reduced local pain scores for abdominal wall endometriosis.
- The procedure demonstrated a favorable safety profile with minor complications.
- Cryoablation has emerged as a minimally invasive alternative to traditional treatments.

**Graphical Abstract:**

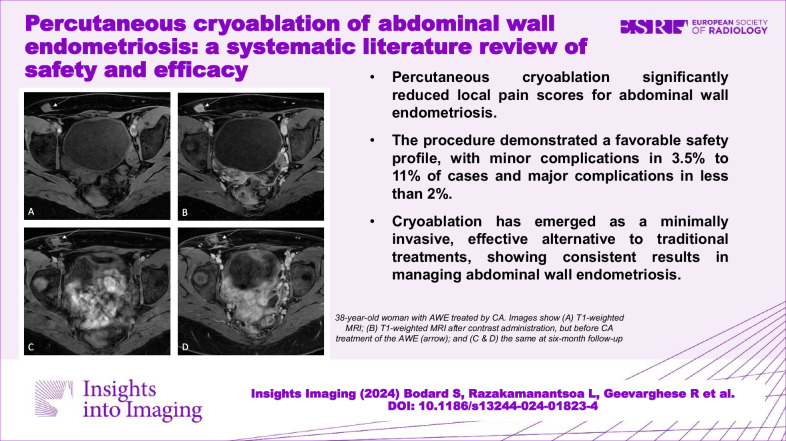

## Introduction

Pelvic endometriosis is a gynecological inflammatory condition related to endometrial tissue-like outside the uterine cavity, with a prevalence of 10% of women in reproductive years [1]. One of the rarest forms of endometriosis is abdominal wall endometriosis (AWE) which is characterized by the abnormal presence of endometrial tissue within the abdominal wall, generally superficial to the peritoneal fascia [2]. Its incidence varies from 0.03% to 3.5%, typically appearing around the age of 35 and often associated with previous cesarean section, occurring in 57% of cases [3, 4]. However, spontaneous parietal nodules have also been observed in women without prior surgical interventions, suggesting a multifaceted etiology for AWE [5]. The predominant symptom of AWE is abdominal pain, significantly impacting the quality of life for those affected [6]. Traditional management strategies for symptomatic AWE typically involve hormonal therapy or surgery [7]. In recent years, percutaneous image-guided cryoablation (CA) has emerged as a promising option to treat soft-tissue lesions, particularly when conventional medical management is ineffective or not preferred by patients [8]. Whatever cryoagent (argon gas vs. liquid nitrogen) or type of cryoprobe is used, CA offers several advantages over conventional surgical interventions. Real-time imaging capabilities enable precise probe placement and monitoring of ice ball formation, thereby minimizing the risks associated with skin burns and peritoneal damage [8]. Additionally, CA’s ability to conform to the contours of the lesion, even in challenging anatomical locations, contributes to its effectiveness. The integration of imaging modalities further enhances precision by ensuring accurate targeting of soft tissue [9–11]. Initial short-term outcomes of CA for AWE published in 2014 have shown promising safety and efficacy profiles, confirmed by subsequent mid- and long-term studies that indicate significant and sustained pain reduction [8, 12]. This systematic review of studies published over the last decade aimed to comprehensively evaluate the safety and efficacy of CA for the management of AWE.

## Methods

A systematic review of the literature was undertaken through PubMed, Embase, Cochrane Library, and Web of Science. The search spanned from the earliest publication on this topic in 2014 to relevant papers published as recently as March 2024.

The search strategy employed a comprehensive approach, utilizing keywords related to AWE (“cesarean scar endometriosis,” “endometriosis node,” “abdominal wall endometriosis,” “abdominal wall endometrioma,” “incisional endometriosis,” “scar endometrioma,” “cutaneous endometriosis,” “nodular endometriosis,” “extrapelvic endometrioma,”) and the specific intervention (“cryoablation”). Studies were included if they (1) featured a retrospective or prospective design; (2) explored CA of AWE; and (3) reported performance metrics of safety and/or efficacy (e.g., assessment of effectiveness in tumor response, pain response, tumor volume reduction, adverse events (AEs)). Exclusions were applied for (1) studies offering only abstracts; (2) non-English language studies; (3) non-human studies; (4) editorial-style reviews; (5) abstracts and posters; (6) conference papers; and (7) case reports or studies with fewer than 3 procedures.

Data extraction was conducted by two independent reviewers using a standardized form encompassing author details, publication year, study type, employed techniques, number of patients, patient demographics (age), follow-up duration, treatment efficacy outcomes (tumor volume decrease, local pain relief using Visual Analog Scale (VAS), and local pain-free survival, referring to the period during which the patient remains free from pain after the intervention), and AEs graded following the guidelines of the Society of Interventional Radiology (SIR) [13] and the National Cancer Institute Common Terminology Criteria Adverse Events (CTCAE, version 4.0).

The methodological quality of the included studies was evaluated using established tools tailored to study design, and employing the Newcastle-Ottawa Scale for cohort studies [14].

Data synthesis was conducted narratively, and provided an overview of treatment techniques, patient characteristics, and outcomes. A subgroup analysis, differentiating between surgical and nonsurgical treatments, aimed to explore variations in efficacy and AEs. The risk of bias in individual studies was assessed to gauge the internal validity of the findings. Potential biases, such as selection bias, information bias, and publication bias, were considered during result interpretation.

**Fig. 1:**
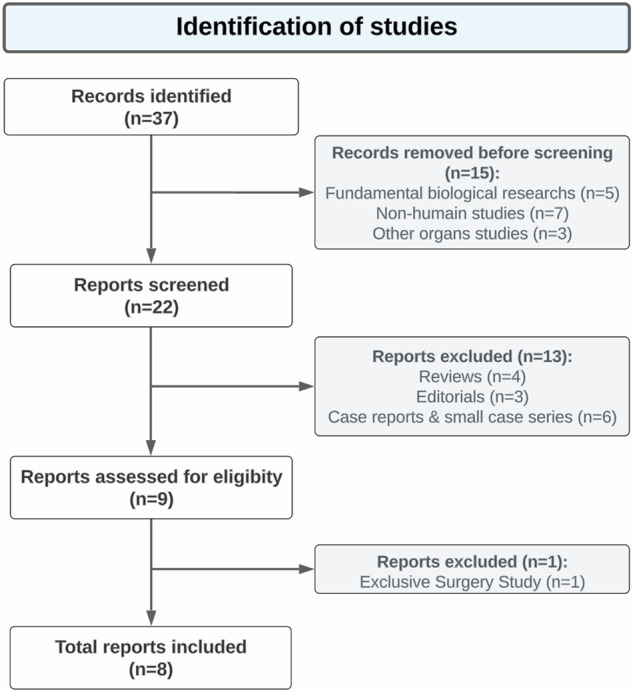
Study flowchart

A qualitative synthesis and descriptive analysis were employed. The review adhered to the Preferred Reporting Items for Systematic Reviews and Meta-Analyses guidelines [15], with registration in NIHR-PROSPERO (CRD 42024505263). Figure 1 presents a flowchart depicting the study selection. Continuous variables were reported as median and 95% confidence interval, while categorical variables were presented as both numbers and percentages (%). Statistical analyses utilized GraphPad Software version 10.0 (Boston, MA), employing chi-square or Fisher’s exact tests for categorical data comparison. Significance was established at a *p*-value < 0.05.

## Results

This review encompassed eight studies, all of which were retrospective [16–23]. One study included a comparison with surgical procedures [18]. The median sample size across these studies was 23 patients (interquartile range (IQR): 6.3–38.5, average: 23, range: 3–42), with a median age of 36.9 years (IQR: 35.6–37). The follow-up period varied from 6 to 60 months. Table 1 summarizes the characteristics of the included studies.

**Table 1:** Studies included in the systematic review

Authors (ref#)	Technique	Study type	No. of patients	Age (median) (IQR)	Follow-up (month) (range)
Cornelis et al [17]	CA	R	4	34.5 (28–39)	1, 3, 6
Maillot et al [18]	CA vs. surgery	Comp /R	7 vs. 13	35.7 (28–39) vs. 31.9 (24–41)	22.5 (6–42) vs. 54 (14–149)
Dibble et al [19]	CA	R	3	40 (37–43)	6–12
Smith et al [20]	CA	R	18	36.9 (30–46)	85 days (median)
Najdawi et al [21]	CA	R	42	37 (33–39.5)	13.5 (1.1–37.7)
Jouffrieau et al [22]	CA	R	29	N/A	6
Bachou et al [23]	CA	R	38	35.5 (IQR: 32–39, range: 24–48)	6 M for all; 12 M for 18 of them (47%)
Marcelin et al [16]	CA	R	40	37 (32–40)	40.5 (26.5–47.2)

*R* retrospective, *Comp* comparative, *CA* cryoablation, *IQR* interquartile range, *SD* standard deviation

The studies consistently demonstrated statistically significant efficacy, with a notable reduction in local pain levels. Local pain scores decreased from a median of 8/10 (IQR 7–9) on the VAS to 1/10 (IQR 0–2) at the last follow-up (*p* < 0.0001). Median complete local pain response rates ranged from 80% [16] to 100% [19], with median local pain-free survival rates reaching 76.8% (IQR 55.3–83.8) at the longest follow-up [16]. Notably, no patient reported a post-procedure VAS score higher than their pre-cryoablation score. In the single study comparing CA and surgery, Maillot et al [18] reported similar effectiveness between the two techniques, with median symptom-free survival rates of 100% at 12 months and 66.7% (95% CI, 5.4; 94.5) at 24 months following CA, compared to 92% (IQR 55.3; 98.9) after surgery at both intervals (*p* = 0.45). Additionally, the median duration for both procedure and hospitalization was shorter after CA (41.5 min (IQR 24–66) and 0.8 days (IQR 0–1), respectively) than after surgery (73.5 min (IQR 35–160) and 2.8 days (IQR 1–12), respectively (both *p* = 0.01)).

In terms of AEs, the studies indicated minor complications (such as cutaneous edema and skin anesthesia) in 3.5% to 11% of cases, with major complications occurring in less than 2% of cases (one patient with second-degree skin burn and peritonitis due to a probable small-bowel injury, which required local skin care and 5 days of hospitalization with intravenous antibiotic therapy. Maillot et al [18] observed a higher incidence of AEs in the surgical group, with 3 patients (23.1%) experiencing severe complications (one parietal defect, one abscess requiring a new intervention 2 weeks after surgery and 4 days of hospitalization complicated by severe chronic pelvic pain; one huge hematoma, followed by a parietal defect lasting a few months) and 9 cases (69.2%) of esthetic scars after surgery, compared to none after CA (*p* = 0.05).

Table 2 summarizes the efficacy and safety results of the procedures.

**Table 2:** Results of studies evaluating procedure efficacy and safety

Authors (ref#)	Efficacy	Adverse events
Cornelis et al [17]	*Local pain response (VAS):* M0 = 6.5; M6 = 1.7 *Tumor volume decrease:* M6 = 85.7% (72.2–100)	None > CTAE 2
Maillot et al [18]	*Local pain response (VAS):* *CA:* M0 = 8.3 (6–10); M1 = 2 (0–4); M6 = 1.9 (0–4) *Surgery:* M0 = 6.7 (3–10); M1 = 3.3 ± 0.10; M6 = 0.7 (0–10) *Tumor volume decrease (mm*^*3*^*):* *CA:* M0 = 3.7 (0.3–11); M6 = 0.5 (0–1.3)	CA: None Surgery: (3/13, 23.1%) severe, (9/13, 69.2%) esthetic
Dibble et al [19]	*Local pain response:* 100% M6 = VAS 0 and 2; M12 = VAS 0	(3/3) acetaminophen or hydromorphone oral
Smith et al [20]	*Local pain response:* 93%	(2/18, 11%), self-limited inflammatory response
Najdawi et al [21]	*Local pain-free survival (VAS):* M6 = 93.8%; M12 = 82.5%	(3/42, 7.1%) minor AE, (1/42, 2%) severe AE
Jouffrieau et al [22]	*Local pain decrease* (%): M1 = 62.1%; M6 = 72.4% *Tumor volume decrease* (%): M6 = 72.4%	(1/29, 3.5%) (minor complication)
Bachou et al [23]	*Local pain relief*: M6 = 31/38 (82%); M12 = 15/18 (83%) *Local pain decrease (median VAS score):* before: 7 (IQR: 6, 8; range: 3–10); M3 = 0 (IQR: 0, 5; range; 0–8) (*p* < 0.001); M6 = 0 (IQR: 0, 1; range; 0–10) (*p* < 0.001); 12 M = 0 (IQR: 0, 2; range: 0–7) (*p* < 0.001) *Tumor volume decrease*: statistically significant at M6 (*p* < 0.001)	None > CTAE 2
Marcelin et al [16]	*Local pain relief*: complete at M3 = 80% (32/40), correlated with the absence of residual endometriosis nodules on MRI *Median pain-free survival rate*: M36: 89.2% (70.1–96.4); M60 = 76.8% (55.3–83.8)	None > CTAE 2

*AE* adverse event, *VAS* visual analog scale, *CA* cryoablation, *CTAE* Common Terminology Criteria for Adverse Events, *M* months

## Discussion

AWE presents a significant challenge in management due to its impact on quality of life and limited treatment options [6]. While hormonal therapy or surgery has traditionally been employed [7], percutaneous image-guided CA has emerged as a promising alternative, particularly in cases where standard approaches fail or are unsuitable [8].

Studies have consistently demonstrated CA’s effectiveness in achieving local control of AWE in the short-, mid-, and long-term. Long-term outcomes are comparable to those achieved with surgical interventions [16] and other noninvasive techniques, such as high-intensity focused ultrasound [24]. While high-intensity focused ultrasound is associated with low recurrence rates, CA may be preferable due to its real-time monitoring of ice build-up, which minimizes collagen matrix damage and its associated tissue granulation—ultimately leading to enhanced long-term esthetic results [20]. Comparisons with other percutaneous techniques, such as radiofrequency and microwave ablation (MWA), highlight CA’s favorable safety profile [25, 26]. While these alternative methods have shown promising pain responses in case reports, the lack of imaging visibility during the procedure and the coagulation of tissue by heat increases the risk of AEs [27]. Indeed, CA forms ice crystals within the tissue, leading to cell death while preserving the surrounding collagen matrix. It offers real-time imaging capabilities that enable precise probe placement and ice ball monitoring, minimizing risks such as collateral damage and contributing to its high safety profile and effectiveness. On the other hand, RFA uses high-frequency current to generate heat, causing tissue necrosis. While widely used and effective, it can cause thermal damage to adjacent tissues due to less precise heat control, leading to higher rates of AEs. Similarly, MWA employs electromagnetic waves to produce heat, resulting in faster ablation times and higher temperatures. However, like RFA, MWA risks uncontrolled heat spread and lacks real-time visualization. Another risk of RFA and MWA is the development of unsightly retractile scars. The choice of technique should consider patient characteristics and lesion size. Future prospective trials are needed to establish evidence-based guidelines for AWE management and refine the selection of the most appropriate ablation method [23, 25–27].

The unique mechanisms of CA, involving ice crystal formation without compromising vital structures like collagen, set it apart from thermal ablation. Procedural intricacies, such as cryoprobe size and choice of cryogenic agent, contribute to tailored treatment outcomes [9–11]. One advantage of CA is its tolerance, as it offers quicker recovery times and reduced hospital stays. Collaborative efforts with gynecology departments, particularly those operating outpatient clinics, have resulted in many AWE patients only requiring outpatient care. The use of local anesthesia/sedation further facilitates patient comfort during the procedure [10]. Despite these advantages, some patients have reported transient inflammation and discomfort post-cryoablation. The importance of thorough pre-procedural consultations and adequate post-procedural management with anti-inflammatory drugs cannot be overlooked. Additionally, the presence of concomitant deep endometriosis may introduce a selection bias, as patients with more severe diseases may be more frequently referred for treatment of AWE. CA may not be suitable for all AWE patients, and further research is needed to explore its applicability, especially in the early stages of endometriosis and when nodules are deep within the tissues. Indeed, scenarios, where CA is most appropriate, include AWE lesions that do not involve critical underlying structures, such as the digestive tract or bladder, situations in which surgery is preferred. CA can be effectively performed in an outpatient setting under local anesthesia, reducing the need for hospitalization and facilitating faster recovery. Patients with hormone-resistant lesions who respond poorly to hormonal therapy, as well as those for whom side effects are problematic, can benefit from this treatment. Additionally, CA is suitable for patients’ considering pregnancy, as hormonal treatment has a contraceptive effect. It is also ideal for patients who are contraindicated for surgery due to medical comorbidities or who prefer a minimally invasive option [1, 28].

This literature review acknowledges limitations due to its reliance on small, single-institution cohort studies with varying follow-up periods. Beyond the CRYOENDOMET clinical trial (NCT03627676), prospective, randomized, controlled, and well-powered trials with more extended follow-up are needed to assess optimal therapeutic management of AWE [21]. These must include a comparison of minimally invasive procedures against or in combination with current medical and surgical therapeutic measures.

In conclusion, percutaneous image-guided CA presents as a promising and minimally invasive option for AWE management. Its distinct advantages, technical considerations, and favorable safety profile highlight its potential for achieving effective and durable outcomes. As research in this field progresses, prospective clinical trials are imperative to establish CA as a new standard treatment for women suffering from AWE.

## Data Availability

Data is available from the authors upon reasonable request.

## Abbreviations

AEs: Adverse events
AWE: Abdominal wall endometriosis
CA: Cryoablation
IQR: Interquartile range
MWA: Microwave ablation
VAS: Visual analog scale
